# Cerebral blood flow in a tri-ethnic population: insights from pCASL perfusion MRI

**DOI:** 10.1007/s00330-025-12160-5

**Published:** 2025-12-03

**Authors:** Rafael Rehwald, Magdalena Sokolska, Carole H. Sudre, Lorna Smith, Therese Tillin, David Atkinson, Nishi Chaturvedi, Alun D. Hughes, Hans Rolf Jäger

**Affiliations:** 1 Neuroradiological Academic Unit, Department of Translational Neuroscience and Stroke, UCL Queen Square Institute of Neurology, London, UK; 2 Department of Radiology, University of Cambridge, Cambridge, UK; 3 Department of Medical Physics and Biomedical Engineering, University College London Hospitals National Health Service (NHS) Foundation Trust, London, UK; 4 MRC Unit for Lifelong Health and Ageing at UCL, Institute of Cardiovascular Science, University College London, London, UK; 5 Dementia Research Centre, UCL Queen Square Institute of Neurology, University College London, London, UK; 6 School of Biomedical Engineering, King’s College, London, UK; 7 Centre for Medical Imaging, Division of Medicine, University College London, London, UK; 8 Lysholm Department of Neuroradiology, National Hospital for Neurology and Neurosurgery, Queen Square, Holborn, London, UK

**Keywords:** Spin labels, Artefacts, Cerebrovascular circulation, Brain, Ethnicity

## Abstract

**Objectives:**

Arterial transit artefacts (ATAs) on pseudo-continuous arterial spin labelling (pCASL) MRI represent visual markers of delayed arterial transit. This study aimed to investigate their prevalence and distribution and to evaluate the effects of sex, ethnicity, intracranial arterial anatomy, and cardiovascular parameters in a subgroup of the UK tri-ethnic population-based Southall and Brent REvisited (SABRE) study.

**Materials and methods:**

We analysed 360 participants—120 each of White European, South Asian, and African Caribbean origin—from the prospective SABRE cohort who underwent 3.0-T brain MRI and clinical assessment between 2014 and 2018. ATAs were visually rated across 40 predefined brain regions on pCASL perfusion images and summarised as percentage ATA scores. Intracranial arterial anatomy was classified on time-of-flight MR angiography, and cardiovascular parameters were obtained from clinical assessment. ATAs were compared by sex and ethnicity, and associations with demographic, anatomical, and cardiovascular factors were analysed using multivariable regression.

**Results:**

Of 360 participants, 284 (78.89%; mean age 70.12 ± 6.58 years; range 49–89; 139 women) had usable pCASL data. ATA prevalence varied across vascular territories and between women and men. African Caribbean participants showed a higher frequency of ATAs in the posterior circulation, whereas in most anterior territories they had fewer ATAs than White Europeans or South Asians.

**Conclusion:**

Visual rating of ATAs revealed sex- and ethnicity-specific differences in ATA distribution, reflecting variations in arterial transit time influenced by intracranial vascular anatomy and cardiovascular parameters. These findings highlight the potential of ATAs as imaging markers for personalised cerebrovascular assessment and risk stratification.

**Key Points:**

***Question*** *Prevalence and distribution of arterial transit artefacts (ATAs) on arterial spin labelling MRI, and their relationship to sex, ethnicity, vascular anatomy, and cardiovascular parameters, have not been systematically investigated.*

***Findings*** *ATAs were most prevalent in African Caribbeans, particularly in MCA–PCA borderzones and PCA territories; no significant differences were found between White Europeans and South Asians.*

***Clinical relevance*** *Visual rating revealed substantial differences in the ATA distribution among ethnic populations, as well as between women and men. Recognising these specific patterns can help distinguish physiological from pathological perfusion, thereby enhancing diagnostic accuracy and treatment planning.*

**Graphical Abstract:**

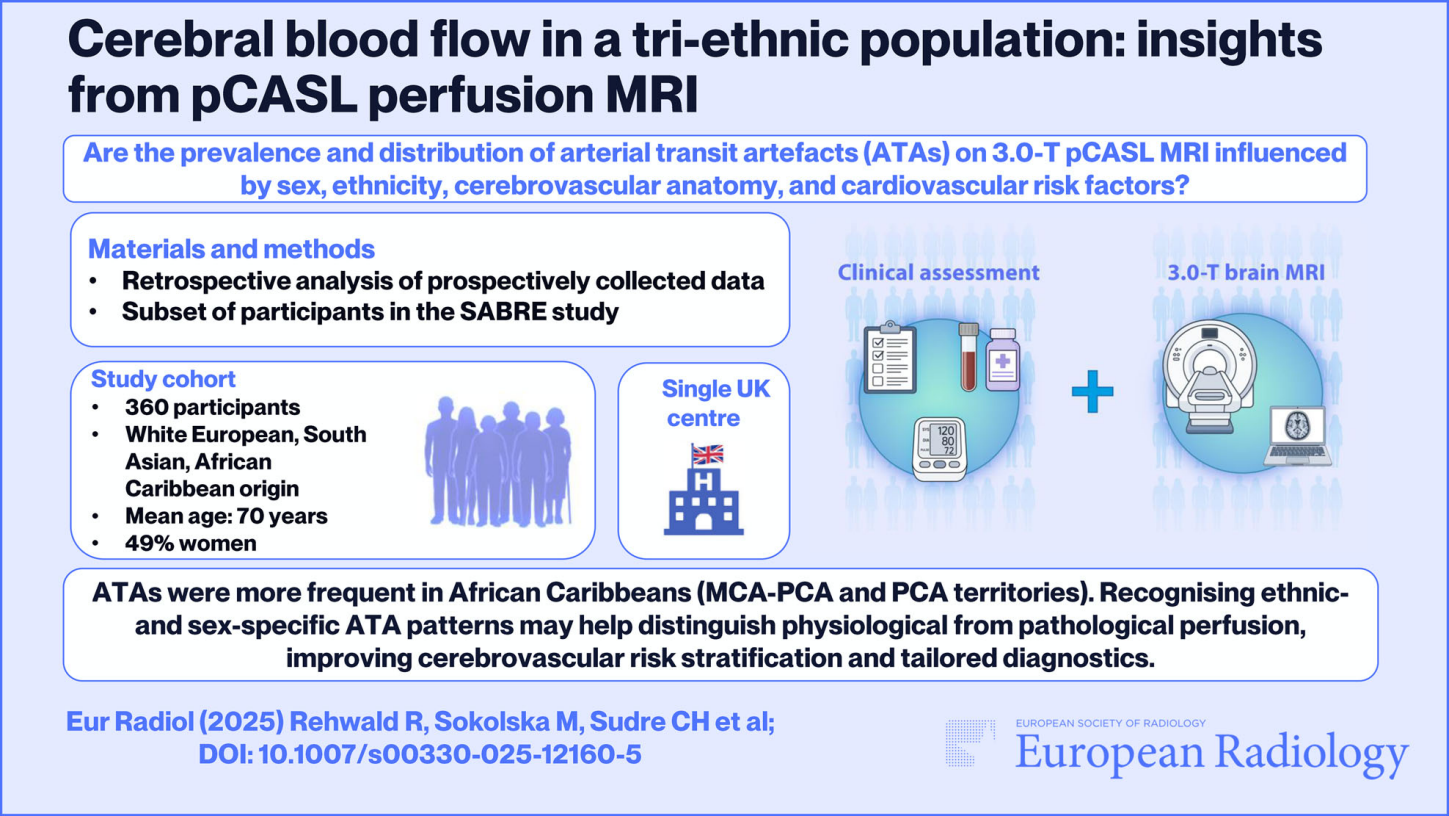

## Introduction

Arterial spin labelling (ASL) is a non-contrast MRI technique relying on magnetically labelled arterial blood water protons as an endogenous tracer for visualisation and quantification of brain perfusion. The consensus paper of the ISMRM Perfusion Study Group and the European ASL in Dementia consortium recommends pseudo-continuous ASL (pCASL) with a single post-labelling delay (PLD) as the current method of choice [1–4]. Like other ASL sequences, pCASL is prone to artefacts due to insufficient labelling efficiency, motion, or prolonged arterial transit time (ATT) [5]. When ATT exceeds the PLD, labelled spins remain fully or partially in the arteries before reaching the cortex, where they appear as serpiginous hyperintensities. This phenomenon, described as arterial transit artefact (ATA), may be seen in one or more vascular territories depending on the underlying mechanism [2, 5–7]. A common spatial distribution of ATAs, known as the borderzone sign, is characterised by serpiginous high signal overlying the cortical borderzones with signal loss in the underlying cortex [3]. Anatomical variations in the circle of Willis (CoW) influence blood flow routing and the arrival of labelled spins, with recent evidence demonstrating ethnic differences in CoW configuration [8, 9]. Variability in ATT between individuals and across brain regions can be affected by several factors, including reduced cardiac output, extra- and intracranial steno-occlusive disease, and advancing age [2, 6, 10–12]. ATAs, which indicate prolonged ATT, are therefore increasingly recognised as important physiological and haemodynamic markers [2, 3, 11, 13]. From a technical perspective, ATA detection also depends on pulse-sequence parameters, particularly the PLD [2].

Few studies have systematically examined the prevalence of ATAs in older adults or elucidated the physiological mechanisms underlying age-related differences in ATT [6, 14, 15]. Ethnic variations in cerebrovascular anatomy, particularly in the CoW [9], and differences in cardiovascular risk factors may further influence ATT and ATA occurrence, yet these factors remain largely unexplored. We therefore aimed to comprehensively assess ATA prevalence and spatial distribution on pCASL imaging in a tri-ethnic cohort from the population-based Southall and Brent REvisited (SABRE) study, using the recommended PLD of 2000 ms for individuals older than 70 years [2, 16]. We investigated the effects of sex, ethnicity, cerebral arterial anatomy, and cardiovascular parameters on the presence of ATAs, to provide new insights into their role as markers of cerebrovascular physiology and haemodynamics.

## Materials and methods

### Study population

**Fig. 1:**
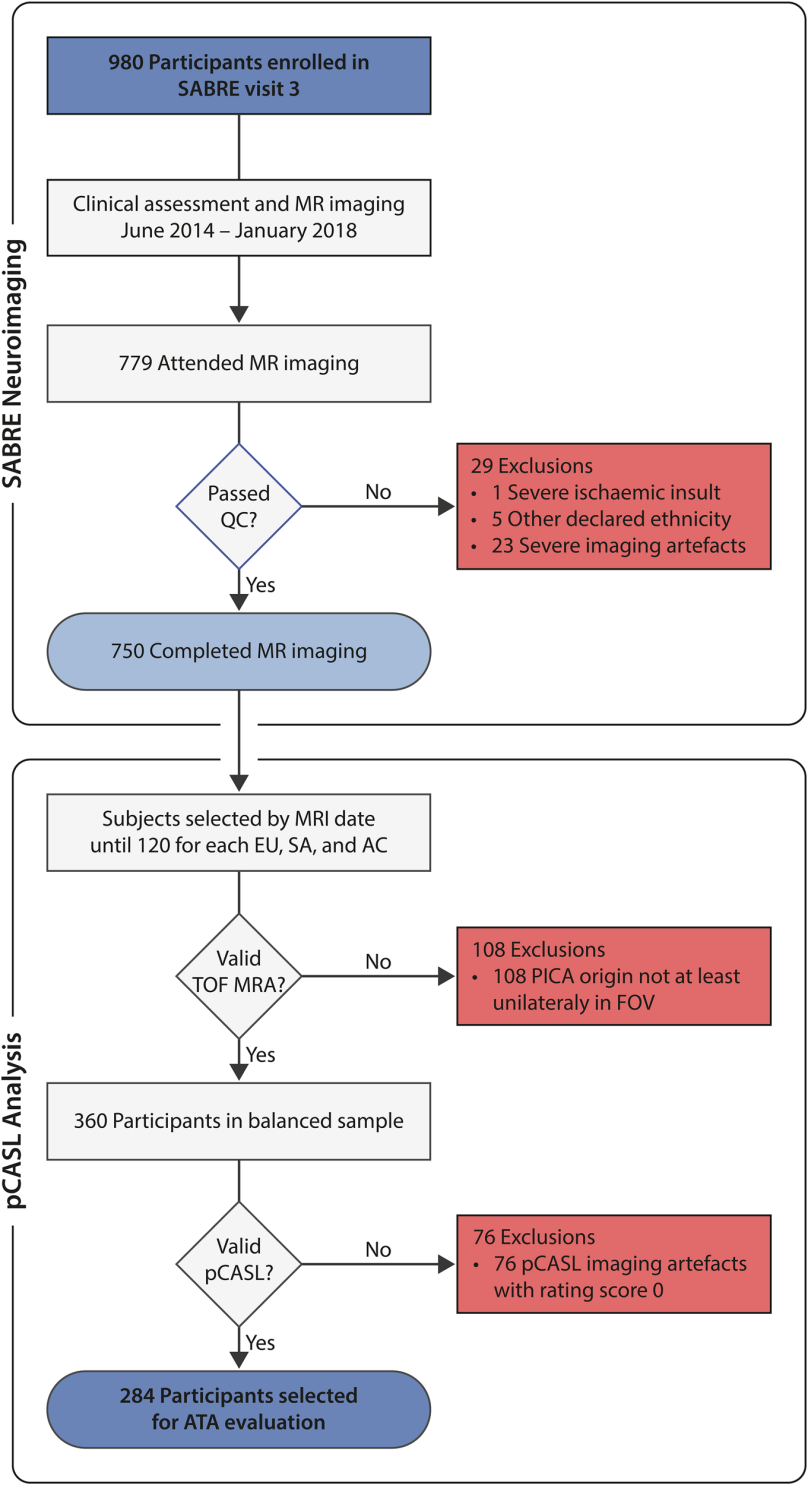
Flowchart outlining the inclusion and exclusion criteria and resulting study cohort for the pCASL ATA analysis. QC, quality control; EU, White European; SA, South Asian; AC, African Caribbean; PICA, posterior inferior cerebellar artery; TOF MRA, time-of-flight MR angiography; FOV, field of view

Sociodemographic, clinical, and neuroradiological data were obtained from the UK-based SABRE study, a population-based investigation of cardiovascular disease among participants of self-identified European, South Asian, and African Caribbean origin [16]. Ethical approval was obtained from the London Fulham Research Ethics Committee (reference number 14/LO/108). A balanced subsample of 360 participants, 120 per ethnic group, was selected chronologically based on vertebrobasilar vascular landmarks; inclusion required the posterior inferior cerebellar artery (PICA) origin to be visible unilaterally in the time-of-flight (TOF) MR angiography (MRA) field of view (FOV). Inclusion and exclusion criteria are detailed in Fig. 1.

### MR imaging

All MR scans were acquired in a single clinical centre, University College London Hospitals (UCLH) NHS Foundation Trust, using a 3.0-T whole-body MRI system (3.0-T Achieva, Philips Healthcare) fitted with an 8-channel SENSE Head phased-array receiver coil (SENSE Head 8, Philips Healthcare).

#### MR angiography

TOF MR angiography was performed with a 3D gradient echo sequence acquiring 140 axial slices over a 200 × 200 × 70 mm FOV (RL × AP × FH). The acquired voxel size was 0.70 × 0.40 × 0.50 mm, reconstructed to a 560 × 560 in-plane matrix. The TR/TE was 25/3.5 ms, nominal flip angle 20°, RF spoiling was used for T1-weighted contrast, and the sequence duration was 6:00 min.

#### Arterial spin labelling

Whole-brain ASL imaging was performed using a pCASL sequence with 2D multi-slice single-shot echo-planar imaging readout with a 240 × 240 × 119 mm FOV (RL × AP × FH), acquiring 20 axial slices with a 1 mm slice gap, a voxel size of 3.75 × 3.75 × 5.00 mm, a TR/TE of 4615/15 ms, a 90° flip angle, and an in-plane matrix size of 80 × 80. The label duration was set to 1800 ms with a PLD of 2000 ms. A total of 35 label/control pairs were acquired, resulting in an acquisition time of 5:32 min. The pCASL labelling plane was positioned perpendicular to the internal carotid arteries based on a phase-contrast MRA survey.

**Fig. 2:**
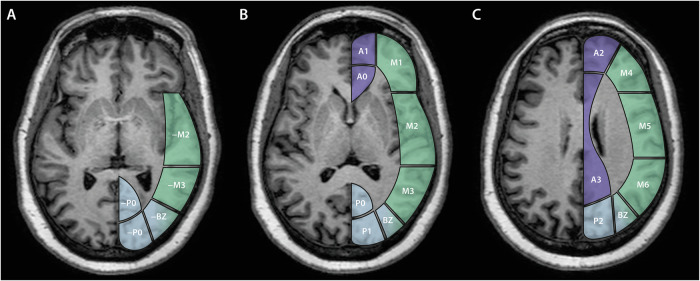
Brain region template used to evaluate cerebral blood flow and the presence of ATAs on pCASL imaging at (**A**) the level inferior to the basal ganglia, (**B**) the level of the basal ganglia, and (**C**) the level superior to the basal ganglia. Cortical vascular territories are shown in violet for the ACA, green for the MCA, and blue for the PCA

**Fig. 3:**
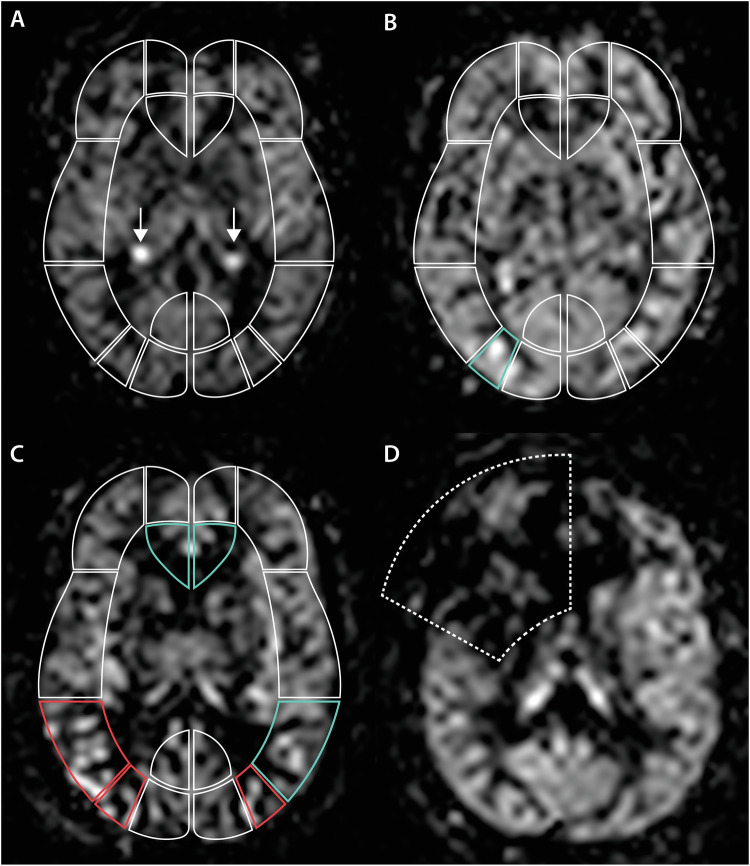
Representative pCASL images from the SABRE study illustrating the ATA–based blood flow rating scale. **A** Normal scan with every brain region scored as normal flow (score 3); arrows highlight the highly perfused choroid plexus. **B** Example showing moderate ATA (score 2) localised in the right posterior borderzone (green), with all other regions rated as normal flow (score 3). **C** Example of severe ATA (score 1) in the posterior borderzone and M3 region of the right hemisphere and in the posterior borderzone in the left hemisphere (red). Bilateral A2 regions and M3 in the left hemisphere rated as moderate ATA (score 2). **D** Example of an excluded ASL scan (score 0) due to a severe coil artefact (dashed outline)

The pCASL sequence was rated visually for ATAs across predefined vascular ROIs at three axial levels using a four-point rating scale (0–3), with a score of ‘0’ for absent or minimal ASL signal, including imaging artefacts, ‘1’ for severe ATA, ‘2’ for moderate ATA, and ‘3’ for normal ASL signal, as previously described [7]. We expanded a previously published ATA rating system, which was originally based on the cortical regions of the Alberta Stroke Programme Early Computed Tomography Score (ASPECTS), to include assessments at the ganglionic and supraganglionic levels [7]. Specifically, we defined additional bilateral cortical vascular borderzone regions at the ganglionic and supraganglionic levels, between M3 and P1 (M3P1 BZ) and M6 and P2 (M6P2 BZ). Furthermore, we added an infraganglionic level, with regions -P0 to -M2, mirroring regions P0 to M2 at the level of the basal ganglia (Fig. 2). In total, 40 brain regions were evaluated across the left and right cerebral hemispheres: 10 inferior to the basal ganglia—approximately at the level of the Sylvian fissure—16 at the level of the basal ganglia, and 14 superior to the basal ganglia, as indicated by the partial volume effect of the basal ganglia. The complete regional template is shown in Fig. 2, with examples presented in Fig. 3. All ATA ratings were performed by R.R. (10 years’ experience). A subsample comprising 10% of the study population was reviewed by H.R.J. (35 years’ experience).

#### ATA score

To evaluate cerebral blood flow (CBF) from regional ATA ratings, we calculated an aggregate ATA percentage score across all vascular territories and levels as follows:

$${ATA\; score}\;( \% )=\left(\frac{a-40}{120-40}\right)$$

where *a* represents the sum of the raw ATA ratings (each ≥ 1) across all 40 predefined brain regions. Within this rating system, the theoretical minimum (*a* = 40) corresponds to severe ATA in every region (ATA score = 0%), and the maximum (*a* = 120) corresponds to a normal ASL signal in all brain regions (ATA score = 100%).

#### Intracranial arterial anatomy

We assessed CoW variants on TOF MRA source images and 3D maximum-intensity projection reconstructions, classifying them according to previously established criteria [17, 18]. Intracranial arterial stenosis (ICAS) was evaluated on TOF MRA, with significant stenosis defined as 50–99% focal luminal narrowing of major intracranial arteries [19].

### Clinical data

Diabetes mellitus status was determined by any of the following criteria: documented diagnosis or prescription for antidiabetic therapy in primary care records; self-reported diagnosis or use of antidiabetic medication; fasting plasma glucose or oral glucose tolerance test results according to WHO guidelines [20], or an HbA₁c > 47 mmol/mol measured at the research clinic. Hypertension was defined as a physician diagnosis, self-reported history, or use of antihypertensive medication. Blood pressure (BP, mm Hg) was measured in the seated position after 5 min of rest in accordance with the 2013 European Society of Hypertension/European Society of Cardiology guidelines [21] using an Omron MIT Elite Plus blood pressure monitor (OMRON Healthcare Co., Ltd.). Pulse pressure (PP, mm Hg) was defined as the difference between resting systolic and diastolic BP. Carotid-to-femoral pulse wave velocity (PWV) was determined with a VICORDER device (SMT medical GmbH & Co. KG) and calculated as the distance between the common carotid and common femoral arteries divided by the pulse transit time (m/s). Echocardiography was performed using an EPIQ 7 ultrasound machine equipped with an X5-1 xMATRIX cardiac probe (Philips Healthcare). Left ventricular ejection fraction (LVEF, %) was calculated as the stroke volume—end-diastolic volume minus end-systolic volume—divided by the end-diastolic volume × 100.

### Statistical analysis

Data were expressed as counts with percentages, or as mean ± standard deviation, as appropriate. Categorical variables were compared with Pearson’s chi-squared test, continuous, normally distributed variables with Student’s *t*-test, and ordinal variables with the Wilcoxon rank-sum test, stratified by sex and ethnicity. Interrater reliability of the ATA ratings was assessed in a stratified random subsample of 10% of the study population, sampled in a 2:1 ratio of usable to excluded MRI scans. Agreement on the exclusion decision was evaluated as a binary outcome using percent agreement, and agreement among usable cases was evaluated using percent agreement and quadratic-weighted Cohen’s κ. All measures were reported with 95% confidence intervals (CI).

To investigate the potential determinants of the ATA score, a series of linear regression models was fitted. Model 1 was adjusted for sex, age, and ethnicity. The morphological classification of the anterior and posterior CoW was included in model 2. Models 3 and 4 were further adjusted for diabetes and hypertension status, respectively. LVEF, PP, and PWV were included stepwise in models 5, 6, and 7, with the fully adjusted model 8 additionally assessing the effect of ICAS. Multivariate ordinal logistic regression models were used to evaluate the combined effects of sex, age, ethnicity, cardiovascular risk factors (hypertension, diabetes, ICAS, PP), and posterior CoW anatomy on raw ATA ratings in the posterior circulation. A two-sided *p* < 0.05 was considered to indicate statistical significance. Statistical analyses were performed with Stata (version 18.5; Stata Corp) and R (version 4.4.1; R Foundation).

## Results

### Participant characteristics

Of the 360 participants selected, 284 (78.89%) had usable pCASL data available; inclusion and exclusion criteria are outlined in Fig. 1. Demographic, anatomical, and clinical characteristics are presented overall and by sex and ethnicity in Tables 1 and 2, with ICAS locations detailed in Supplementary Table 1.

**Table 1:** Participant characteristics, overall, and by sex and ethnicity

		Total			White European			South Asian			African Caribbean		
		*N*	Mean ± SD	Range	*N*	Mean ± SD	Range	*N*	Mean ± SD	Range	*N*	Mean ± SD	Range
Age (years)	Total	284	70.12 ± 6.58	49–89	99	71.52 ± 5.48	56–85	85	70.56 ± 5.99	49–85	100	68.52 ± 7.68	54–89
	Women	139	68.07 ± 6.97	49–89	34	69.53 ± 6.05	56–83	38	68.16 ± 6.30	49–81	67	67.28 ± 7.70	54–89
	Men	145	72.20 ± 5.50	57–85	65	72.57 ± 4.89	65–85	47	72.51 ± 4.99	65–85	33	71.03 ± 7.13	57–84
LVEF (%)	Total	219	66.92 ± 8.58	33.01–86.52	75	65.14 ± 9.24	33.00–81.17	68	67.68 ± 7.99	45.87–86.52	76	68.00 ± 8.23	49.04–83.01
	Women	109	67.91 ± 8.21	47.81–86.52	30	65.67 ± 8.16	48.38–80.37	28	69.54 ± 8.26	47.81–86.52	51	68.32 ± 8.09	49.04–83.01
	Men	110	65.94 ± 8.85	33.01–82.83	45	64.78 ± 9.97	33.01–81.17	40	66.38 ± 7.62	45.87–80.71	25	67.34 ± 8.64	51.60–82.83
PWV (m/s)	Total	266	11.50 ± 3.36	6.76–39.17	92	11.81 ± 3.09	7.84–32.16	80	11.74 ± 2.55	7.20–22.44	94	10.99 ± 4.09	6.76–39.17
	Women	133	11.02 ± 2.77	6.76–22.44	33	11.47 ± 2.48	8.01–19.82	36	11.25 ± 2.52	7.20–22.44	64	10.66 ± 3.04	6.76–21.60
	Men	133	11.97 ± 3.80	7.04–39.17	59	11.99 ± 3.40	7.84–32.16	44	12.13 ± 2.54	8.20–20.30	30	11.69 ± 5.75	7.04–39.17
Pulse pressure (mm Hg)	Total	284	61.42 ± 12.55	32.00–98.50	99	58.44 ± 10.57	32.00–98.50	85	64.52 ± 13.60	34.50–96.50	100	62.23 ± 12.99	35.50–95.50
	Women	139	61.35 ± 13.29	32.00–95.50	34	58.10 ± 10.78	32.00–80.50	38	63.96 ± 13.67	34.50–94.50	67	61.51 ± 14.04	37.00–95.50
	Men	145	61.49 ± 11.85	35.50–98.50	65	59.38 ± 10.52	41.00–98.50	47	64.97 ± 13.67	41.00–96.50	33	60.67 ± 10.71	35.50–78.00
ATA score (%)	Total	284	82.28 ± 8.50	47.50–100.00	99	83.00 ± 8.20	47.50–100.00	85	83.43 ± 9.16	56.25–100.00	100	80.58 ± 8.00	56.25–96.50
	Women	139	84.05 ± 8.13	56.25–100.00	34	85.18 ± 7.95	66.25–100.00	38	86.81 ± 8.01	67.50–100.00	67	81.90 ± 7.81	56.25–96.25
	Men	145	80.58 ± 8.52	47.50–97.50	65	81.87 ± 8.16	47.50–96.25	47	80.69 ± 9.20	56.25–97.50	33	77.88 ± 7.82	61.25–91.25

*LVEF* left ventricular ejection fraction, *PWV* pulse wave velocity, *ATA* arterial transit artefact

**Table 2:** Cardiovascular risk factors, overall, and by sex and ethnicity

		Total			White European			South Asian			African Caribbean		
		*N*	Prevalence	%	*N*	Prevalence	%	*N*	Prevalence	%	*N*	Prevalence	%
Diabetes	Total	282	57	20.21	99	10	10.10	85	21	24.71	98	26	26.53
	Women	137	33	24.09	34	4	11.76	38	10	26.32	65	19	29.23
	Men	145	24	16.55	65	6	9.23	47	11	23.40	33	7	21.21
Hypertension	Total	240	131	54.58	99	35	35.35	85	59	69.41	56	37	66.07
	Women	104	53	50.96	34	9	26.47	38	23	60.53	32	21	65.62
	Men	136	78	57.35	65	26	40.00	47	36	76.60	24	16	66.67
ICAS > 50%	Total	284	14	4.93	99	5	5.05	85	6	7.06	100	3	3.00
	Women	139	5	3.60	34	1	2.94	38	2	5.26	67	2	2.99
	Men	145	9	6.21	65	4	6.15	47	4	8.51	33	1	3.03

*ICAS* intracranial arterial stenosis

### Interrater reliability

Agreement on the exclusion decision was 97.22% (95% CI, 85.83–99.51). Among usable pCASL sequences, agreement in ATA ratings was 89.90% (95% CI, 87.83–91.65), with a quadratic-weighted Cohen’s κ of 0.86 (95% CI, 0.83–0.89), indicating excellent interrater reliability [22].

### Circle of Willis

The detailed classification of CoW variants is provided in Supplementary Tables 2 and 3. Overall, variant frequencies were similar between men and women and across ethnic groups; however, African Caribbean men had the highest prevalence of a bilateral fetal-type posterior cerebral artery (PCA). Among the 284 participants with a usable pCASL sequence, 200 (70.42%) had an incomplete posterior CoW, and 84 (29.58%) had a complete posterior CoW. The posterior CoW was more frequently complete in women (*N* = 49; 35.25%) than in men (*N* = 35; 24.14%; *p* = 0.04).

### Arterial transit artefacts

ATA ratings varied substantially across vascular territories. Territories supplied by the PCA and middle cerebral artery (MCA) bilaterally, as well as the intermediate borderzone region, showed increased ATT relative to the other brain regions, while the anterior ACA territories at the level of the basal ganglia were found to have a normal perfusion signal. ATA prevalence was highest superior to the basal ganglia. Table 1 presents ATA scores overall and by sex and ethnicity. Women had significantly higher scores than men (*p* < 0.01). African Caribbeans showed lower ATA scores compared with White Europeans and South Asians, with trends toward significance (*p* = 0.07 and *p* = 0.13, respectively).

**Fig. 4:**
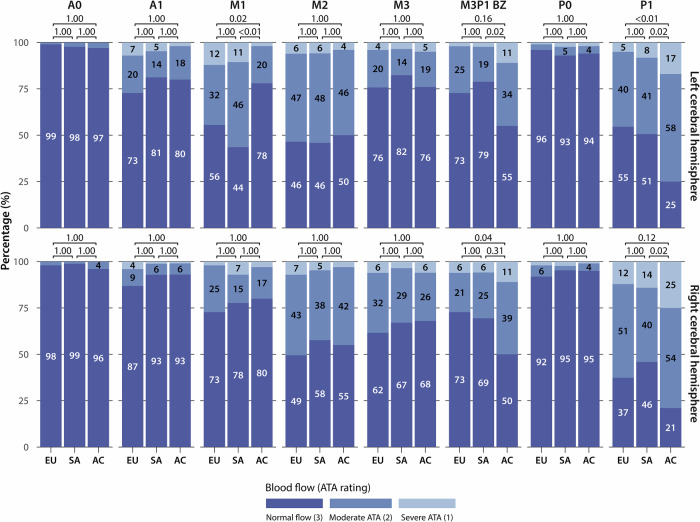
Bar plots showing the percentage distribution of ATA ratings for each brain region in the left (upper panel) and right (lower panel) cerebral hemispheres at the level of the basal ganglia by ethnicity (White European [EU], *N* = 99; South Asian [SA], *N* = 85; African Caribbean [AC], *N* = 100). Percentages of ATA scores ≥ 3 are shown (rounded); groupwise comparisons use Holm-adjusted *p*-values

In relation to ethnicity, notable disparities were observed across the study cohort. In general, African Caribbean individuals showed more ATAs in most posterior territories and in the MCA–PCA borderzones at the level of the basal ganglia and inferior level compared to White European and South Asian individuals. In the MCA territories, the opposite trend was observed: in the most anterior region, African Caribbeans were found to have fewer ATAs than individuals of White European or South Asian origin. A detailed summary of ATA ratings for each region, both overall and by ethnic group, along with the corresponding group comparisons, is shown in Fig. 4 and Supplementary Figs. 1 and 2.

### Linear regression analysis

Across all linear regression models (Supplementary Table 4), age and African Caribbean ethnicity were consistently associated with a lower ATA score. Male sex was found to be significant in models 1 to 4, though this effect was less attenuated and non-significant after adjustment for cardiovascular risk factors in models 5 to 8. Among anterior CoW variants, only variant h (unilateral absent or hypoplastic A1 with a single contralateral A1 supplying both post-communicating ACAs) was linked to the ATA score in models 2 to 4 [9, 17]; this effect was, however, much less attenuated and non-significant after adjusting for PP, PWV, and intracranial stenosis. In the posterior circulation, CoW variant ‘g’ (unilateral fetal-type PCA with absence of the contralateral posterior communicating artery) was associated with an approximately 5% increase in the ATA score across all models [9, 17]. Elevated PP was a significant predictor of a higher ATA score in models 6 to 8, whereas other cardiovascular risk factors showed no statistically significant effects. ICAS was negatively associated with the ATA score, suggesting lower ATA ratings in participants with intracranial stenosis, but this relationship did not reach statistical significance.

### Multivariate ordinal logistic regression analysis

In the posterior circulation across all three axial levels, male sex and African Caribbean ethnicity were significantly associated with lower ATA ratings, indicative of delayed arterial transit. Age significantly affected ATA ratings only at the supraganglionic level. At the same level, a bilateral fetal-type PCA was positively associated with higher ATA ratings, indicating shorter transit times. Cardiovascular risk factors were not significantly associated with ATA ratings in any model. Full regression results are presented in Supplementary Tables 5–7.

## Discussion

In this analysis, which evaluated ATA ratings across 40 brain regions at three anatomical levels, significant differences in ATA prevalence were observed both across regions and between ethnic groups. African Caribbean individuals had more ATAs bilaterally in the PCA territories and in the MCA–PCA borderzones and lower ATA scores overall than White European and South Asian participants. These findings were consistent across women and men, whereas no substantial differences were found between White European and South Asian participants. Linear regression confirmed a strong association between African Caribbean ethnicity and a reduced ATA score.

Healthy individuals exhibit substantial variability in cerebral perfusion, driven by multiple physiological parameters [10]. A prolonged ATT, which manifests as a reduced ATA score, can result from any process that slows blood flow velocity or increases the path length between the labelling and imaging planes [12, 14]. Because ATT is inherently longest in distal vascular branches and vascular borderzones, the regional ATA distribution observed in this analysis is consistent with cerebrovascular anatomy [23–25].

Age-associated prolongation of ATT has been attributed to structural micro- and macrovascular remodelling, including increased vascular tortuosity in large arteries and arterioles, causing an increase in pathlength and thereby delaying the blood arrival time [14, 15, 26, 27]. These findings align with our results, which demonstrated a significant association between ATT and age across all ATA score models. Notably, in the posterior circulation, the link between ATA and age was particularly significant in the level superior to the basal ganglia. Overall, our findings indicate a decreasing ATA score with advancing age. However, the ethnic disparities observed are unlikely to reflect only differences in age or age-related effects, as the African Caribbean participants were significantly younger than the White European and South Asian individuals in this cohort.

Barkeij Wolf et al [8] reported alterations in quantified CBF secondary to morphological variation of the posterior CoW using pCASL perfusion imaging with a PLD of 1525 ms, showing that a unilateral fetal-type PCA causes an apparent ipsilateral CBF increase and an apparent decrease in the ipsilateral cerebellar CBF. These imaging abnormalities, resulting in ipsilateral pseudo-hyperperfusion and contralateral pseudo-hypoperfusion images, may be clinically misinterpreted [28]. Zaharchuk et al [3] have described the borderzone sign, a characteristic and common artefactual ASL signal bilaterally in the anterior and particularly in the posterior MCA borderzones. However, because distal vascular territories inherently have longer ATT, reduced ATA scores in those regions may reflect delayed arrival rather than true perfusion deficits [2].

Previous studies have demonstrated increased CBF and shorter ATT in women [14, 29, 30]. The mechanisms responsible for sex differences in ATT remain incompletely understood, but may include lower haematocrit in women, associated with both reduced oxygen-carrying capacity, which may be compensated by higher CBF, and lower blood viscosity, resulting in increased flow velocity and in shortened ATT [14, 31, 32]. Our findings align with these observations and might extend beyond sex differences, partially explaining the differences observed among the three ethnic groups. Moreover, haematocrit is linked to cardiovascular risk factors such as obesity and diabetes risk, which, in view of sex and ethnically divergent risk factor susceptibility, might partially explain the ATT differences observed [33]. Consequently, lower ATA scores in men—both overall and regionally—could reflect higher cumulative cardiovascular risk and its adverse impact on arterial transit [34].

The linear relationship between elevated PP and higher ATA scores may reflect the dual role of PP as a marker of both cardiac function and arterial stiffening. Ageing and vascular risk factors increase arterial stiffening, leading to a widened PP, an independent marker for cardiovascular disease [35–37]. Structural and functional arterial wall remodelling reduces mural elasticity and increases stiffness, thereby amplifying pulsatile pressure and flow transmission in the cerebral vasculature, manifesting, for example, as white matter hyperintensities [35, 38]. Furthermore, rising systolic BP, the key factor driving the widening of the PP, has been positively associated with increased flow velocity in the MCA [36, 39].

In this study, prolonged ATT was observed overall and, in particular, among African Caribbean participants in the posterior circulation and borderzone regions, consistent with previous findings. Hendrikse et al [23] attributed this finding to the unique posterior circulation geometry, where blood in the PCAs flows downstream, parallel to the imaging plane, while Mutsaerts et al [15] linked the heterogeneity of the ATT in the posterior circulation to the high variability in the intracranial posterior arterial anatomy, as shown for example by Wentland et al [40]. However, in this study, no constant attenuated effect of the vascular geometry on the ATT in posterior cerebral vascular territories could be confirmed, although in the linear regression analysis, posterior CoW type ‘g’ was found to significantly reduce ATAs.

Variation in arterial diameter and blood flow velocity between men and women and across ethnic groups may also contribute to the observed differences in ATA ratings [14, 41]. However, the biological and mechanical mechanisms regulating vascular remodelling and blood flow are complex [42], and genetic factors may further influence CBF. For example, Wierenga et al (2013) demonstrated the differential effects of the apolipoprotein E genotype on CBF, which had previously been shown to vary across ethnic populations and geographical latitudes [43–45].

A key strength of this study is the large tri-ethnic population-based cohort, the availability of comprehensive anthropometric and clinical data, and the use of a standardised 3.0-T MRI scanning protocol. Using the currently recommended PLD, we conducted the first systematic analysis of ATA frequency based on visual rating, revealing substantial differences between ethnic populations and differences in ATT depending on the intracranial arterial anatomy.

This study has some limitations. The SABRE cohort did not include younger participants, and it is therefore difficult to generalise the present findings to all age groups. In the multivariate ordinal regression models, McFadden’s pseudo *R*^2^ (*ρ*^2^) ranged from 0.11 to 0.24. Although *ρ*^2^ inherently assumes lower values than the more common *R*^2^ metric, these results indicate limited model strength and warrant cautious interpretation.

Inherent to the 2D pCASL acquisition used in this study is a slight, incremental increase of the PLD with each slice acquired, resulting in a minimally longer PLD in the superior slices, compared to an equivalent 3D pCASL acquisition [46–48]. A potential effect of this slight PLD increase is a reduction of observed ATAs compared to 3D acquisition. However, it is important to note that the observed differences in ATA prevalence between acquisition schemes may not be solely attributable to PLD. For example, 3D GRASE, a spin-echo–based readout, inherently attenuates intravascular signal due to its reduced sensitivity to flowing spins. Consequently, comparisons between 2D and 3D acquisitions may conflate the effects of PLD with those arising from differences in readout design. Attention to both PLD timing and readout design is therefore essential when interpreting ATAs in ASL data.

In accordance with the ISMRM Perfusion Study Group and European ASL in Dementia Consortium guidelines, a 2000-ms PLD was applied for healthy individuals older than 70 years [2]. Recently, sequences using multi-PLD or time-encoded ASL imaging sequences have become available, which potentially would have allowed for direct quantification of the ATT [2, 49]. Overall, factors influencing ATT, such as age and sex, compare well with results from previous investigations. Detailed ethnic differences in ATA patterns have, however, not been previously described. The relative delayed arrival of labelled blood in the posterior circulation of African Caribbean individuals represents a novel finding, with the exact underlying mechanisms requiring further study.

Differences in ATA by sex and ethnicity likely reflect the interplay of multiple factors beyond physiological baseline ATT variability, including intracranial vascular geometry, cardiovascular parameters, and genetic predisposition. These findings support the use of ATAs as non-invasive pCASL imaging markers to characterise cerebrovascular anatomy and haemodynamics across diverse populations. Clinically, recognising ethnic- and sex-specific ATA distribution patterns may help distinguish normal transit artefacts from true perfusion deficits, enabling more accurate risk stratification, diagnostics, and personalised treatment of cerebrovascular disorders.

## Supplementary information

ELECTRONIC SUPPLEMENTARY MATERIAL

## Abbreviations

ASL: Arterial spin labelling
ATA: Arterial transit artefact
ATT: Arterial transit time
BP: Blood pressure
CoW: Circle of Willis
ICAS: Intracranial arterial stenosis
LVEF: Left ventricular ejection fraction
pCASL: Pseudo-continuous ASL
PLD: Post-labelling delay
PP: Pulse pressure
PWV: Pulse wave velocity
