# Supplementary material for: Cerebral blood flow in a tri-ethnic population: insights from pCASL perfusion MRI
Source: Eur Radiol. 2025 Dec 3;36(5):3854–65. doi: 10.1007/s00330-025-12160-5 (PMC13086786; doi:10.1007/s00330-025-12160-5)
Supplement: Supplementary file 1 — ELECTRONIC SUPPLEMENTARY MATERIAL [file 330_2025_12160_MOESM1_ESM.pdf]

Cerebral blood flow in a tri-ethnic population:
Insights from pCASL perfusion MRI
ELECTRONIC SUPPLEMENTARY MATERIAL

Supplemental Table 1: Location of intracranial stenoses.

	BA	ICA left	ICA right	MCA M1 left	MCA M1 right	MCA M2 left	MCA M2 right	PCA P2 left	PCA P2 right	Total
**Female, EU**	-	-	-	-	-	1	-	-	-	**1**
**Female, SA**	-	1	-	1	-	-	-	-	-	**2**
**Female, AC**	-	-	1	-	1	-	-	-	-	**2**
**Male, EU**	1	-	1	-	-	-	-	1	1	**4**
**Male, SA**	-	1	2	-	-	-	1	-	-	**4**
**Male, AC**	-	-	-	-	1	-	-	-	-	**1**
**Total**	**1**	**2**	**4**	**1**	**2**	**1**	**1**	**1**	**1**	**14**

Note: EU, White European; SA, South Asian; AC, African Caribbean. BA, Basilar artery; ICA, Internal carotid artery; MCA, Middle cerebral artery; PCA, Posterior cerebral artery.

Supplemental Table 2: Anatomical classification of the anterior circle of Willis.

	ACAH	Azg	BiHem ACA	a	b	c	c3	d	e	g	h	h3	Total
**Female, EU**	1	1	–	11	1	3	1	8	–	6	2	–	**34**
**Female, SA**	–	–	1	9	2	4	–	4	1	14	3	–	**38**
**Female, AC**	1	–	1	26	5	1	1	4	1	21	5	1	**67**
**Male, EU**	–	–	1	26	2	4	–	8	2	17	5	–	**65**
**Male, SA**	2	–	–	16	3	3	–	6	–	15	2	–	**47**
**Male, AC**	–	–	–	18	–	4	1	–	1	7	2	–	**33**
**Total**	**4**	**1**	**3**	**106**	**13**	**19**	**3**	**30**	**5**	**80**	**19**	**1**	**284**

Note: EU, White European; SA, South Asian; AC, African Caribbean.
Original classification cited from Krabbe-Hartkamp et al. (1998), expanded by Rehwald et al. (2024)*:

ACAH*: Unilateral anterior cerebral artery hypoplasia.
Azg*: Azygos anterior cerebral artery.
BiHemACA*: Bi-hemispheric anterior cerebral artery.
a: A single anterior communicating artery. The ICA bifurcates into the precommunicating segment of the anterior cerebral artery and the MCA.
b: Two (or more) anterior communicating arteries.
c: Medial artery of the corpus callosum arises from the anterior communicating artery.
c3*: Duplication of the median artery of the corpus callosum.
d: Fusion of the anterior cerebral arteries over a short distance.
e: Anterior cerebral arteries form a common trunk and split distally into two postcommunicating segments.
g: Hypoplasia or absence of an anterior communication.
h: One precommunicating segment of an anterior cerebral artery gives rise to both post-communicating segments of the anterior cerebral arteries.
h3*: Unilateral anterior cerebral artery trifurcation.

Supplemental Table 3: Anatomical classification of the posterior circle of Willis.

	a	*b*	*c*	*d*	*e*	*f*	*g*	*h*	*i*	*j*	*Total*
**Female, EU**	5	3	1	9	9	0	2	2	2	1	**34**
**Female, SA**	2	2	2	12	11	4	4	0	0	1	**38**
**Female, AC**	21	8	5	17	8	3	2	0	0	3	**67**
**Male, EU**	7	6	3	13	24	0	3	6	2	1	**65**
**Male, SA**	4	0	0	17	18	1	3	2	1	1	**47**
**Male, AC**	6	6	3	8	5	0	2	0	1	2	**33**
**Total**	**45**	**25**	**14**	**76**	**75**	**8**	**16**	**10**	**6**	**9**	**284**

Note: EU, White European; SA, South Asian; AC, African Caribbean. Original classification from Krabbe-Hartkamp et al. (1998):

a: Bilateral posterior communicating arteries present.
b: Posterior cerebral artery originates predominantly from the ICA.
This variant is known as a unilateral fetal-type posterior cerebral artery, the posterior communicating artery on the other side is patent.
c: Bilateral fetal-type posterior cerebral arteries with both precommunicating segments of the posterior cerebral arteries patent.
d: Fusion of the anterior cerebral arteries over a short distance.
e: Unilateral posterior communicating artery present.
f: Unilateral fetal-type posterior cerebral artery and hypoplasia or absence of the precommunicating segment of the posterior cerebral artery.
g: Unilateral fetal-type posterior cerebral artery and hypoplasia or absence of the precommunicating segment of the posterior cerebral artery.
h: Unilateral fetal-type posterior cerebral artery and hypoplasia or absence of both a precommunicating segment of the posterior cerebral artery and the posterior communicating artery.
i: Bilateral fetal-type posterior cerebral arteries with hypoplasia or absence of both precommunicating segments of the posterior cerebral arteries.
j: Bilateral fetal-type posterior cerebral arteries with hypoplasia or posterior cerebral artery.

Supplemental Table 4: Linear regression results of the models investigating the association of the ATA score, subject base characteristics, intracranial arterial anatomy and cardiovascular risk factors.

		Model 1			Model 2			Model 3			Model 4			Model 5			Model 6			Model 7			Model 8		
	**Variable**	**OR**	**95% CI**	**p-value**	**OR**	**95% CI**	**p-value**	**OR**	**95% CI**	**p-value**	**OR**	**95% CI**	**p-value**	**OR**	**95% CI**	**p-value**	**OR**	**95% CI**	**p-value**	**OR**	**95% CI**	**p-value**	**OR**	**95% CI**	**p-value**
	Age (years)	-0.222**	[-0.374, -0.0702]	(0.004)	-0.225**	[-0.382, -0.0684]	(0.005)	-0.250**	[-0.411, -0.0878]	(0.003)	-0.275**	[-0.474, -0.0755]	(0.007)	-0.269*	[-0.488, -0.0497]	(0.017)	-0.366**	[-0.592, -0.139]	(0.002)	-0.400**	[-0.645, -0.154]	(0.002)	-0.397**	[-0.643, -0.151]	(0.002)
	Sex: Female†	*0*	*[0, 0]*	*(.)*	*0*	*[0, 0]*	*(.)*	*0*	*[0, 0]*	*(.)*	*0*	*[0, 0]*	*(.)*	*0*	*[0, 0]*	*(.)*	*0*	*[0, 0]*	*(.)*	*0*	*[0, 0]*	*(.)*	*0*	*[0, 0]*	*(.)*
	Sex: Male	-3.633***	[-5.675, -1.592]	(0.001)	-3.488**	[-5.587, -1.388]	(0.001)	-3.552***	[-5.653, -1.451]	(0.001)	-2.936*	[-5.266, -0.607]	(0.014)	-1.263	[-3.861, 1.335]	(0.338)	-1.245	[-3.796, 1.306]	(0.337)	-1.112	[-3.750, 1.525]	(0.406)	-1.099	[-3.740, 1.542]	(0.412)
	Ethnicity: European†	*0*	*[0, 0]*	*(.)*	*0*	*[0, 0]*	*(.)*	*0*	*[0, 0]*	*(.)*	*0*	*[0, 0]*	*(.)*	*0*	*[0, 0]*	*(.)*	*0*	*[0, 0]*	*(.)*	*0*	*[0, 0]*	*(.)*	*0*	*[0, 0]*	*(.)*
	Ethnicity: South Asian	-0.169	[-2.527, 2.189]	(0.888)	-0.238	[-2.697, 2.221]	(0.849)	0.191	[-2.283, 2.665]	(0.879)	0.638	[-2.052, 3.329]	(0.641)	-0.0474	[-3.075, 2.980]	(0.975)	-0.572	[-3.570, 2.425]	(0.707)	0.155	[-2.964, 3.274]	(0.922)	0.119	[-3.005, 3.243]	(0.940)
	Ethnicity: African Caribbean	-4.284***	[-6.641, -1.928]	(0.000)	-4.152**	[-6.672, -1.632]	(0.001)	-3.964**	[-6.498, -1.429]	(0.002)	-3.963*	[-7.069, -0.856]	(0.013)	-4.027*	[-7.444, -0.609]	(0.021)	-4.242*	[-7.601, -0.883]	(0.014)	-3.518	[-7.102, 0.0650]	(0.054)	-3.618*	[-7.214, -0.0221]	(0.049)
	A1H	-	-	-	-1.440	[-9.738, 6.857]	(0.733)	-1.237	[-9.506, 7.032]	(0.769)	-2.809	[-12.52, 6.904]	(0.569)	-2.667	[-12.24, 6.903]	(0.583)	-1.688	[-11.11, 7.737]	(0.724)	-2.756	[-12.25, 6.735]	(0.567)	-2.893	[-12.40, 6.616]	(0.549)
	BiHemACA	-	-	-	0.453	[-9.145, 10.05]	(0.926)	-0.383	[-9.958, 9.191]	(0.937)	-4.157	[-16.20, 7.883]	(0.497)	-5.203	[-22.06, 11.65]	(0.543)	-1.836	[-18.57, 14.90]	(0.829)	-0.996	[-17.79, 15.80]	(0.907)	-0.828	[-17.65, 15.99]	(0.923)
Anterior circle of Willis	a†	-	-	-	*0*	*[0, 0]*	*(.)*	*0*	*[0, 0]*	*(.)*	*0*	*[0, 0]*	*(.)*	*0*	*[0, 0]*	*(.)*	*0*	*[0, 0]*	*(.)*	*0*	*[0, 0]*	*(.)*	*0*	*[0, 0]*	*(.)*
	b	-	-	-	0.814	[-3.926, 5.553]	(0.736)	1.074	[-3.638, 5.786]	(0.654)	2.809	[-3.073, 8.692]	(0.348)	3.138	[-3.142, 9.418]	(0.325)	2.801	[-3.370, 8.971]	(0.371)	2.406	[-3.780, 8.593]	(0.443)	2.357	[-3.839, 8.552]	(0.453)
	c	-	-	-	-0.138	[-4.154, 3.879]	(0.946)	-0.00665	[-4.003, 3.990]	(0.997)	-0.274	[-4.546, 3.999]	(0.900)	1.294	[-3.188, 5.775]	(0.569)	1.109	[-3.293, 5.511]	(0.620)	0.512	[-4.159, 5.183]	(0.829)	0.456	[-4.222, 5.135]	(0.847)
	c3	-	-	-	1.742	[-7.883, 11.37]	(0.722)	1.753	[-7.823, 11.33]	(0.719)	-0.310	[-16.85, 16.23]	(0.971)	0.916	[-15.34, 17.17]	(0.912)	3.244	[-12.81, 19.30]	(0.690)	2.737	[-13.31, 18.78]	(0.736)	2.672	[-13.39, 18.74]	(0.743)
	d	-	-	-	-1.327	[-4.794, 2.139]	(0.451)	-1.419	[-4.869, 2.031]	(0.419)	-1.307	[-4.999, 2.385]	(0.486)	0.160	[-3.794, 4.115]	(0.936)	0.225	[-3.657, 4.108]	(0.909)	-0.256	[-4.349, 3.838]	(0.902)	-0.406	[-4.521, 3.709]	(0.846)
	e	-	-	-	-4.007	[-11.32, 3.310]	(0.282)	-4.350	[-11.64, 2.938]	(0.241)	-5.596	[-13.96, 2.763]	(0.188)	-1.554	[-11.05, 7.947]	(0.747)	-1.501	[-10.83, 7.826]	(0.751)	-1.394	[-10.73, 7.945]	(0.768)	-1.586	[-10.95, 7.777]	(0.738)
	g	-	-	-	-0.485	[-2.879, 1.908]	(0.690)	-0.220	[-2.611, 2.170]	(0.856)	-0.535	[-3.202, 2.132]	(0.693)	0.195	[-2.747, 3.137]	(0.896)	0.160	[-2.728, 3.049]	(0.913)	-0.886	[-3.923, 2.151]	(0.565)	-0.952	[-3.997, 2.093]	(0.538)
	h	-	-	-	4.717*	[0.650, 8.784]	(0.023)	4.812*	[0.676, 8.949]	(0.023)	5.537*	[1.021, 10.05]	(0.016)	5.114	[-0.666, 10.89]	(0.082)	4.207	[-1.508, 9.921]	(0.148)	2.985	[-3.155, 9.126]	(0.338)	2.753	[-3.422, 8.927]	(0.380)
	a†	-	-	-	*0*	*[0, 0]*	*(.)*	*0*	*[0, 0]*	*(.)*	*0*	*[0, 0]*	*(.)*	*0*	*[0, 0]*	*(.)*	*0*	*[0, 0]*	*(.)*	*0*	*[0, 0]*	*(.)*	*0*	*[0, 0]*	*(.)*
	b	-	-	-	-0.384	[-4.429, 3.661]	(0.852)	-0.526	[-4.546, 3.494]	(0.797)	-0.180	[-4.938, 4.579]	(0.941)	1.546	[-3.633, 6.725]	(0.556)	2.560	[-2.580, 7.701]	(0.327)	3.320	[-2.267, 8.907]	(0.242)	3.211	[-2.390, 8.811]	(0.259)
	c	-	-	-	2.613	[-2.366, 7.592]	(0.302)	3.297	[-1.779, 8.373]	(0.202)	2.354	[-3.599, 8.307]	(0.436)	-0.0665	[-6.870, 6.737]	(0.985)	0.951	[-5.770, 7.673]	(0.780)	3.011	[-4.279, 10.30]	(0.416)	2.900	[-4.405, 10.20]	(0.434)
	d	-	-	-	1.035	[-2.098, 4.167]	(0.516)	0.907	[-2.206, 4.020]	(0.567)	0.230	[-3.395, 3.855]	(0.901)	0.0335	[-3.921, 3.988]	(0.987)	0.559	[-3.343, 4.461]	(0.778)	1.186	[-2.964, 5.336]	(0.573)	1.095	[-3.066, 5.255]	(0.604)
Posterior circle of Willis	e	-	-	-	-0.148	[-3.411, 3.115]	(0.929)	-0.331	[-3.576, 2.914]	(0.841)	-0.877	[-4.538, 2.783]	(0.637)	-0.841	[-4.790, 3.109]	(0.675)	-0.0550	[-3.976, 3.866]	(0.978)	1.410	[-2.787, 5.606]	(0.508)	1.445	[-2.758, 5.648]	(0.498)
	f	-	-	-	2.444	[-3.919, 8.808]	(0.450)	-0.327	[-7.140, 6.485]	(0.925)	-1.084	[-8.631, 6.463]	(0.777)	0.905	[-8.012, 9.821]	(0.841)	2.520	[-6.315, 11.36]	(0.574)	3.162	[-5.766, 12.09]	(0.485)	3.159	[-5.780, 12.10]	(0.486)
	g	-	-	-	5.144*	[0.352, 9.936]	(0.035)	5.045*	[0.282, 9.808]	(0.038)	5.402	[-0.117, 10.92]	(0.055)	4.819	[-0.964, 10.60]	(0.102)	5.228	[-0.458, 10.91]	(0.071)	6.134*	[0.339, 11.93]	(0.038)	6.131*	[0.329, 11.93]	(0.038)
	h	-	-	-	2.970	[-2.906, 8.845]	(0.321)	3.127	[-2.713, 8.968]	(0.293)	3.072	[-3.082, 9.225]	(0.326)	2.963	[-3.646, 9.572]	(0.377)	3.591	[-2.914, 10.10]	(0.277)	4.884	[-1.792, 11.56]	(0.150)	4.736	[-1.959, 11.43]	(0.164)
	i	-	-	-	2.837	[-4.255, 9.928]	(0.432)	2.923	[-4.122, 9.968]	(0.415)	2.486	[-4.917, 9.889]	(0.509)	0.726	[-7.149, 8.602]	(0.856)	-0.0535	[-7.807, 7.700]	(0.989)	0.610	[-7.244, 8.463]	(0.878)	0.267	[-7.640, 8.175]	(0.947)
	j	-	-	-	-0.371	[-6.405, 5.663]	(0.904)	-0.931	[-6.948, 5.087]	(0.761)	-1.493	[-8.361, 5.374]	(0.669)	-1.225	[-8.030, 5.580]	(0.723)	-1.346	[-8.027, 5.336]	(0.691)	-0.673	[-7.453, 6.107]	(0.845)	-0.818	[-7.616, 5.980]	(0.812)
	Diabetes: No†	-	-	-	-	-	-	*0*	*[0, 0]*	*(.)*	*0*	*[0, 0]*	*(.)*	*0*	*[0, 0]*	*(.)*	*0*	*[0, 0]*	*(.)*	*0*	*[0, 0]*	*(.)*	*0*	*[0, 0]*	*(.)*
	Diabetes: Yes	-	-	-	-	-	-	-2.120	[-4.598, 0.358]	(0.093)	-1.970	[-4.810, 0.871]	(0.173)	-0.437	[-3.614, 2.741]	(0.786)	-0.889	[-4.027, 2.248]	(0.577)	-1.520	[-4.813, 1.773]	(0.363)	-1.460	[-4.760, 1.841]	(0.383)
	Hypertension: No†	-	-	-	-	-	-	-	-	-	*0*	*[0, 0]*	*(.)*	*0*	*[0, 0]*	*(.)*	*0*	*[0, 0]*	*(.)*	*0*	*[0, 0]*	*(.)*	*0*	*[0, 0]*	*(.)*
	Hypertension: Yes	-	-	-	-	-	-	-	-	-	-0.955	[-3.388, 1.479]	(0.440)	-1.168	[-3.843, 1.508]	(0.390)	-1.400	[-4.032, 1.232]	(0.295)	-1.877	[-4.612, 0.858]	(0.177)	-1.881	[-4.620, 0.857]	(0.177)
	LVEF (%)	-	-	-	-	-	-	-	-	-	-	-	-	0.0589	[-0.0893, 0.207]	(0.434)	0.0589	[-0.0866, 0.204]	(0.425)	0.0786	[-0.0705, 0.228]	(0.299)	0.0849	[-0.0652, 0.235]	(0.265)
	Pulse pressure (mm Hg)	-	-	-	-	-	-	-	-	-	-	-	-	-	-	-	0.153**	[0.0399, 0.267]	(0.008)	0.176**	[0.0587, 0.294]	(0.004)	0.190**	[0.0675, 0.312]	(0.003)
	PWV (m/s)	-	-	-	-	-	-	-	-	-	-	-	-	-	-	-	-	-	-	-0.0710	[-0.450, 0.308]	(0.712)	-0.0779	[-0.458, 0.302]	(0.686)
	ICAS: No†	-	-	-	-	-	-	-	-	-	-	-	-	-	-	-	-	-	-	-	-	-	*0*	*[0, 0]*	*(.)*
	ICAS: Yes	-	-	-	-	-	-	-	-	-	-	-	-	-	-	-	-	-	-	-	-	-	-2.132	[-7.346, 3.081]	(0.420)
	Constant	101.3***	[90.59, 112.0]	(0.000)	100.6***	[89.37, 111.7]	(0.000)	102.6***	[91.05, 114.1]	(0.000)	104.7***	[90.86, 118.6]	(0.000)	98.83***	[78.98, 118.7]	(0.000)	96.18***	[76.59, 115.8]	(0.000)	96.13***	[75.14, 117.1]	(0.000)	95.03***	[73.84, 116.2]	(0.000)
	Observations (N)		284			282			280			238			189			189			176			176	
	R^2		0.112			0.173			0.190			0.217			0.187			0.222			0.240			0.243	
	p-value		0.000			0.000			0.000			0.000			0.070			0.017			0.021			0.025	

95% confidence intervals in brackets

* < 0.05, ** p < 0.01, *** p < 0.001; † base category

<u>Note:</u> LVEF, Left ventricular ejection fraction; PWV, Pulse wave velocity; ICAS, Intracranial arterial stenosis (50–99% focal luminal narrowing)

Supplemental Table 5: Multivariate ordinal regression ATA posterior circulation.
Level: Inferior to the basal ganglia (igl)

Variable: Category: Region	Regression Coef.	Std. Error	Z-value	95% CI (lower)	95% CI (upper)	p-value
Sex: Male: 1	0.023	0.766	0.030	-1.478	1.524	0.976
Sex: Male: 2	-0.983	0.337	-2.914	-1.644	-0.322	0.004
Sex: Male: 3	-1.073	0.379	-2.829	-1.817	-0.330	0.005
Sex: Male: 4	-0.418	1.225	-0.341	-2.820	1.983	0.733
Sex: Male: 5	-0.709	0.339	-2.093	-1.372	-0.045	0.036
Sex: Male: 6	-0.857	0.400	-2.142	-1.642	-0.073	0.032
Ethnicity: South Asian: 1	0.102	0.892	0.114	-1.647	1.850	0.909
Ethnicity: South Asian: 2	-0.201	0.396	-0.509	-0.978	0.575	0.611
Ethnicity: South Asian: 3	0.178	0.435	0.410	-0.675	1.031	0.682
Ethnicity: South Asian: 4	-0.294	1.435	-0.205	-3.106	2.518	0.837
Ethnicity: South Asian: 5	-0.672	0.382	-1.758	-1.421	0.077	0.079
Ethnicity: South Asian: 6	-0.094	0.440	-0.214	-0.956	0.767	0.83
Ethnicity: African Caribbean: 1	-0.443	0.891	-0.497	-2.188	1.303	0.619
Ethnicity: African Caribbean: 2	-0.530	0.421	-1.259	-1.354	0.295	0.208
Ethnicity: African Caribbean: 3	-0.974	0.463	-2.101	-1.882	-0.065	0.036
Ethnicity: African Caribbean: 4	-0.792	1.438	-0.551	-3.610	2.026	0.582
Ethnicity: African Caribbean: 5	-1.432	0.427	-3.358	-2.268	-0.596	0.001
Ethnicity: African Caribbean: 6	-1.605	0.473	-3.392	-2.533	-0.678	0.001
Unilateral fetal PCA: 1	0.088	0.769	0.115	-1.419	1.596	0.909
Unilateral fetal PCA: 2	-0.084	0.402	-0.210	-0.872	0.703	0.834
Unilateral fetal PCA: 3	0.390	0.431	0.906	-0.454	1.235	0.365
Unilateral fetal PCA: 4	-0.502	1.009	-0.498	-2.479	1.475	0.619
Unilateral fetal PCA: 5	0.488	0.409	1.193	-0.314	1.289	0.233
Unilateral fetal PCA: 6	0.467	0.474	0.985	-0.462	1.396	0.325
Bilateral fetal PCA: 1	1.010	1.431	0.705	-1.796	3.815	0.481
Bilateral fetal PCA: 2	-0.278	0.644	-0.432	-1.541	0.984	0.666
Bilateral fetal PCA: 3	-0.114	0.589	-0.193	-1.269	1.041	0.847
Bilateral fetal PCA: 4	10.497	39644.802	0.000	-77691.887	77712.881	1.000
Bilateral fetal PCA: 5	0.117	0.678	0.173	-1.212	1.446	0.863
Bilateral fetal PCA: 6	0.505	0.674	0.749	-0.817	1.826	0.454
ICAS: Yes: 1	-0.162	1.523	-0.107	-3.147	2.822	0.915
ICAS: Yes: 2	0.656	0.775	0.848	-0.862	2.175	0.397
ICAS: Yes: 3	-0.786	0.722	-1.088	-2.201	0.630	0.277
ICAS: Yes: 4	-1.918	1.357	-1.413	-4.578	0.742	0.158
ICAS: Yes: 5	0.588	0.622	0.945	-0.631	1.806	0.345
ICAS: Yes: 6	0.315	0.789	0.400	-1.231	1.862	0.69
Diabetes: Yes: 1	-0.838	0.632	-1.327	-2.077	0.400	0.184
Diabetes: Yes: 2	-0.633	0.389	-1.630	-1.395	0.128	0.103
Diabetes: Yes: 3	-0.205	0.407	-0.504	-1.003	0.593	0.614
Diabetes: Yes: 4	-1.046	0.882	-1.186	-2.775	0.682	0.235
Diabetes: Yes: 5	-0.246	0.385	-0.641	-1.000	0.507	0.522
Diabetes: Yes: 6	0.019	0.436	0.042	-0.835	0.872	0.966
Hypertension: Yes: 1	-0.076	0.629	-0.121	-1.308	1.156	0.904
Hypertension: Yes: 2	-0.243	0.339	-0.715	-0.907	0.422	0.475
Hypertension: Yes: 3	-0.455	0.375	-1.213	-1.189	0.280	0.225
Hypertension: Yes: 4	-0.819	0.983	-0.833	-2.746	1.107	0.405
Hypertension: Yes: 5	-0.014	0.357	-0.039	-0.713	0.685	0.969
Hypertension: Yes: 6	-0.650	0.385	-1.688	-1.404	0.105	0.091
Age (years): 1	-0.053	0.078	-0.688	-0.205	0.099	0.491
Age (years): 2	-0.028	0.031	-0.909	-0.089	0.033	0.364
Age (years): 3	-0.066	0.033	-1.971	-0.132	0.000	0.049
Age (years): 4	-0.049	0.087	-0.568	-0.220	0.121	0.57
Age (years): 5	-0.041	0.029	-1.401	-0.098	0.016	0.161
Age (years): 6	-0.043	0.038	-1.132	-0.119	0.032	0.258
Pulse pressure (mm Hg): 1	0.057	0.037	1.553	-0.015	0.129	0.12
Pulse pressure (mm Hg): 2	0.010	0.015	0.660	-0.020	0.040	0.509
Pulse pressure (mm Hg): 3	0.021	0.018	1.162	-0.014	0.056	0.245
Pulse pressure (mm Hg): 4	0.069	0.056	1.223	-0.041	0.179	0.221
Pulse pressure (mm Hg): 5	0.014	0.014	0.990	-0.013	0.041	0.322
Pulse pressure (mm Hg): 6	0.011	0.018	0.600	-0.024	0.046	0.548

Note: The respective base categories of each variable are: Sex: Female; Hypertension: No; ICAS: No; Posterior CoW: Normal PCA; Ethnicity: White European; Diabetes: No.
Brain regions: 1, igl_P0_r; 2, igl_P1_r; 3, igl_M3P1_BZ_r; 4, igl_P0_l; 5 igl_P1_l; 6 igl_M3P1_BZ_l.
ICAS, Intracranial arterial stenosis (50–99% focal luminal narrowing); PCA, Posterior cerebral artery.

Supplemental Table 6: Multivariate ordinal regression ATA posterior circulation.
Level: Basal ganglia (gl)

Variable: Category: Region	Regression Coef.	Std. Error	Z-value	95% CI (lower)	95% CI (upper)	p-value
Sex: Male: 1	-0.354	0.906	-0.391	-2.129	1.420	0.696
Sex: Male: 2	-0.635	0.360	-1.764	-1.341	0.071	0.078
Sex: Male: 3	-0.949	0.418	-2.270	-1.768	-0.130	0.023
Sex: Male: 4	-0.964	1.228	-0.785	-3.370	1.442	0.432
Sex: Male: 5	-1.063	0.361	-2.946	-1.770	-0.356	0.003
Sex: Male: 6	-0.521	0.437	-1.193	-1.378	0.335	0.233
Ethnicity: South Asian: 1	0.231	1.100	0.210	-1.924	2.386	0.834
Ethnicity: South Asian: 2	0.273	0.375	0.726	-0.463	1.008	0.468
Ethnicity: South Asian: 3	-0.094	0.444	-0.212	-0.964	0.776	0.832
Ethnicity: South Asian: 4	-1.304	1.084	-1.203	-3.428	0.820	0.229
Ethnicity: South Asian: 5	-0.221	0.397	-0.558	-1.000	0.557	0.577
Ethnicity: South Asian: 6	0.408	0.485	0.841	-0.543	1.360	0.400
Ethnicity: African Caribbean: 1	-0.197	1.290	-0.153	-2.726	2.332	0.879
Ethnicity: African Caribbean: 2	-0.965	0.457	-2.113	-1.860	-0.070	0.035
Ethnicity: African Caribbean: 3	-1.482	0.521	-2.843	-2.504	-0.460	0.004
Ethnicity: African Caribbean: 4	-1.516	1.382	-1.097	-4.225	1.193	0.273
Ethnicity: African Caribbean: 5	-1.628	0.444	-3.671	-2.498	-0.759	0.000
Ethnicity: African Caribbean: 6	-1.169	0.535	-2.183	-2.218	-0.119	0.029
Unilateral fetal PCA: 1	0.207	1.142	0.181	-2.030	2.445	0.856
Unilateral fetal PCA: 2	0.297	0.381	0.780	-0.449	1.043	0.435
Unilateral fetal PCA: 3	0.549	0.468	1.173	-0.368	1.466	0.241
Unilateral fetal PCA: 4	0.989	1.654	0.598	-2.253	4.231	0.550
Unilateral fetal PCA: 5	0.193	0.370	0.522	-0.532	0.919	0.602
Unilateral fetal PCA: 6	0.734	0.519	1.414	-0.284	1.752	0.157
Bilateral fetal PCA: 1	-0.420	1.343	-0.313	-3.052	2.212	0.755
Bilateral fetal PCA: 2	-0.117	0.650	-0.180	-1.391	1.158	0.857
Bilateral fetal PCA: 3	0.805	0.714	1.128	-0.594	2.205	0.259
Bilateral fetal PCA: 4	0.791	1.591	0.497	-2.327	3.909	0.619
Bilateral fetal PCA: 5	0.151	0.567	0.267	-0.959	1.262	0.790
Bilateral fetal PCA: 6	0.996	0.717	1.390	-0.408	2.401	0.164
ICAS: Yes: 1	-1.071	1.857	-0.577	-4.711	2.569	0.564
ICAS: Yes: 2	0.445	0.686	0.648	-0.900	1.790	0.517
ICAS: Yes: 3	-0.205	0.687	-0.299	-1.552	1.142	0.765
ICAS: Yes: 4	-3.362	1.377	-2.441	-6.061	-0.663	0.015
ICAS: Yes: 5	0.942	0.703	1.341	-0.435	2.320	0.180
ICAS: Yes: 6	-0.489	0.816	-0.599	-2.089	1.111	0.549
Diabetes: Yes: 1	-0.554	1.074	-0.516	-2.658	1.550	0.606
Diabetes: Yes: 2	-0.842	0.434	-1.940	-1.693	0.008	0.052
Diabetes: Yes: 3	-0.429	0.447	-0.960	-1.306	0.447	0.337
Diabetes: Yes: 4	0.390	1.223	0.319	-2.006	2.786	0.750
Diabetes: Yes: 5	-0.638	0.406	-1.571	-1.434	0.158	0.116
Diabetes: Yes: 6	-0.511	0.461	-1.109	-1.414	0.392	0.267
Hypertension: Yes: 1	-0.133	0.903	-0.148	-1.903	1.636	0.883
Hypertension: Yes: 2	-0.396	0.352	-1.126	-1.086	0.294	0.260
Hypertension: Yes: 3	-0.377	0.389	-0.972	-1.139	0.384	0.331
Hypertension: Yes: 4	-0.272	1.133	-0.240	-2.493	1.950	0.811
Hypertension: Yes: 5	-0.040	0.347	-0.114	-0.720	0.641	0.909
Hypertension: Yes: 6	-0.459	0.419	-1.094	-1.280	0.363	0.274
Age (years): 1	-0.073	0.078	-0.937	-0.225	0.079	0.349
Age (years): 2	-0.058	0.030	-1.926	-0.117	0.001	0.054
Age (years): 3	-0.040	0.036	-1.096	-0.111	0.031	0.273
Age (years): 4	-0.120	0.093	-1.296	-0.301	0.061	0.195
Age (years): 5	-0.019	0.032	-0.573	-0.082	0.045	0.567
Age (years): 6	-0.076	0.037	-2.080	-0.149	-0.004	0.038
Pulse pressure (mm Hg): 1	0.087	0.051	1.695	-0.014	0.188	0.090
Pulse pressure (mm Hg): 2	0.018	0.015	1.188	-0.012	0.048	0.235
Pulse pressure (mm Hg): 3	0.010	0.018	0.529	-0.026	0.046	0.597
Pulse pressure (mm Hg): 4	0.102	0.056	1.818	-0.008	0.212	0.069
Pulse pressure (mm Hg): 5	-0.008	0.016	-0.468	-0.039	0.024	0.640
Pulse pressure (mm Hg): 6	0.024	0.019	1.295	-0.012	0.061	0.195

Note: The respective base categories of each variable are: Sex: Female; Hypertension: No; ICAS: No; Posterior CoW: Normal PCA; Ethnicity: White European; Diabetes: No.
Brain regions: 1, gl_P0_r; 2, gl_P1_r; 3, gl_M3P1_BZ_r; 4, gl_P0_l; 5, gl_P1_l; 6, gl_M3P1_BZ_l
ICAS, Intracranial arterial stenosis (50–99% focal luminal narrowing); PCA, Posterior cerebral artery

Supplemental Table 7: Multivariate ordinal regression ATA posterior circulation.
Level: Superior to the basal ganglia (sgl)

Variable: Category: Region	Regression Coef.	Std. Error	Z-value	95% CI (lower)	95% CI (upper)	p-value
Sex: Male: 1	-0.424	0.316	-1.340	-1.044	0.196	0.180
Sex: Male: 2	-0.964	0.375	-2.569	-1.699	-0.228	0.010
Sex: Male: 3	-0.775	0.324	-2.393	-1.409	-0.140	0.017
Sex: Male: 4	-0.819	0.494	-1.659	-1.787	0.149	0.097
Ethnicity: South Asian: 1	0.312	0.354	0.881	-0.382	1.005	0.378
Ethnicity: South Asian: 2	-0.321	0.431	-0.746	-1.165	0.523	0.456
Ethnicity: South Asian: 3	0.124	0.369	0.336	-0.599	0.848	0.737
Ethnicity: South Asian: 4	-0.658	0.545	-1.207	-1.727	0.410	0.227
Ethnicity: African Caribbean: 1	-0.928	0.407	-2.281	-1.726	-0.131	0.023
Ethnicity: African Caribbean: 2	-0.678	0.443	-1.530	-1.546	0.191	0.126
Ethnicity: African Caribbean: 3	-1.247	0.433	-2.882	-2.095	-0.399	0.004
Ethnicity: African Caribbean: 4	-0.641	0.638	-1.005	-1.891	0.609	0.315
Unilateral fetal PCA: 1	-0.215	0.380	-0.566	-0.961	0.530	0.571
Unilateral fetal PCA: 2	0.464	0.446	1.040	-0.410	1.338	0.298
Unilateral fetal PCA: 3	-0.447	0.375	-1.194	-1.181	0.287	0.233
Unilateral fetal PCA: 4	1.586	0.751	2.113	0.115	3.057	0.035
Bilateral fetal PCA: 1	1.425	0.582	2.447	0.284	2.566	0.014
Bilateral fetal PCA: 2	0.918	0.626	1.465	-0.310	2.146	0.143
Bilateral fetal PCA: 3	1.273	0.559	2.276	0.177	2.370	0.023
Bilateral fetal PCA: 4	1.702	1.332	1.278	-0.908	4.313	0.201
ICAS: Yes: 1	0.427	0.611	0.699	-0.770	1.625	0.484
ICAS: Yes: 2	-0.173	0.655	-0.264	-1.456	1.111	0.792
ICAS: Yes: 3	-1.051	0.658	-1.598	-2.340	0.238	0.110
ICAS: Yes: 4	1.040	1.290	0.807	-1.488	3.568	0.420
Diabetes: Yes: 1	-0.591	0.376	-1.573	-1.328	0.145	0.116
Diabetes: Yes: 2	0.132	0.435	0.303	-0.721	0.985	0.762
Diabetes: Yes: 3	-0.278	0.393	-0.708	-1.047	0.492	0.479
Diabetes: Yes: 4	-0.777	0.546	-1.423	-1.847	0.293	0.155
Hypertension: Yes: 1	0.262	0.341	0.769	-0.406	0.930	0.442
Hypertension: Yes: 2	-0.124	0.378	-0.328	-0.866	0.617	0.743
Hypertension: Yes: 3	-0.100	0.330	-0.304	-0.746	0.546	0.762
Hypertension: Yes: 4	-0.156	0.483	-0.323	-1.103	0.791	0.747
Age (years): 1	-0.076	0.028	-2.743	-0.131	-0.022	0.006
Age (years): 2	-0.084	0.032	-2.653	-0.146	-0.022	0.008
Age (years): 3	-0.032	0.028	-1.150	-0.087	0.023	0.250
Age (years): 4	-0.108	0.041	-2.650	-0.187	-0.028	0.008
Pulse pressure (mm Hg): 1	0.016	0.013	1.176	-0.010	0.042	0.240
Pulse pressure (mm Hg): 2	0.038	0.018	2.148	0.003	0.073	0.032
Pulse pressure (mm Hg): 3	0.015	0.013	1.153	-0.011	0.042	0.249
Pulse pressure (mm Hg): 4	0.046	0.023	1.998	0.001	0.091	0.046

Note: The respective base categories of each variable are: Sex: Female; Hypertension: No; ICAS: No; Posterior CoW: Normal PCA; Ethnicity: White European; Diabetes: No.
Brain regions: 1, sgl_P2_r; 2, sgl_M6P2_BZ_r; 3, sgl_P2_l; 4, sgl_M6P2_BZ_l
ICAS, Intracranial arterial stenosis (50–99% focal luminal narrowing); PCA, Posterior cerebral artery

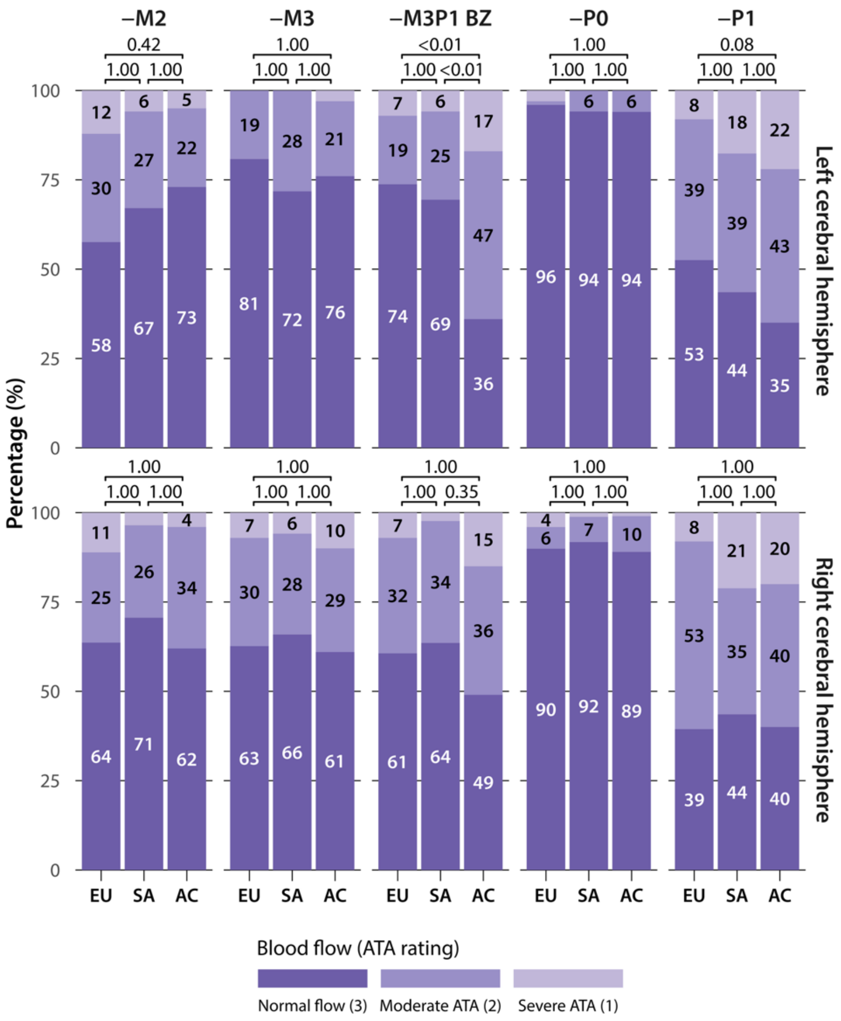

Supplemental Figure 1: Bar plots showing the percentage distribution of ATA ratings for each brain region in the left (upper panel) and right (lower panel) cerebral hemispheres at the level inferior to the basal ganglia by ethnicity (White European [EU], N = 99; South Asian [SA], N = 85; African Caribbean [AC], N = 100). Percentages of ATA scores ≥3 are shown (rounded); groupwise comparisons use Holm-adjusted p-values.

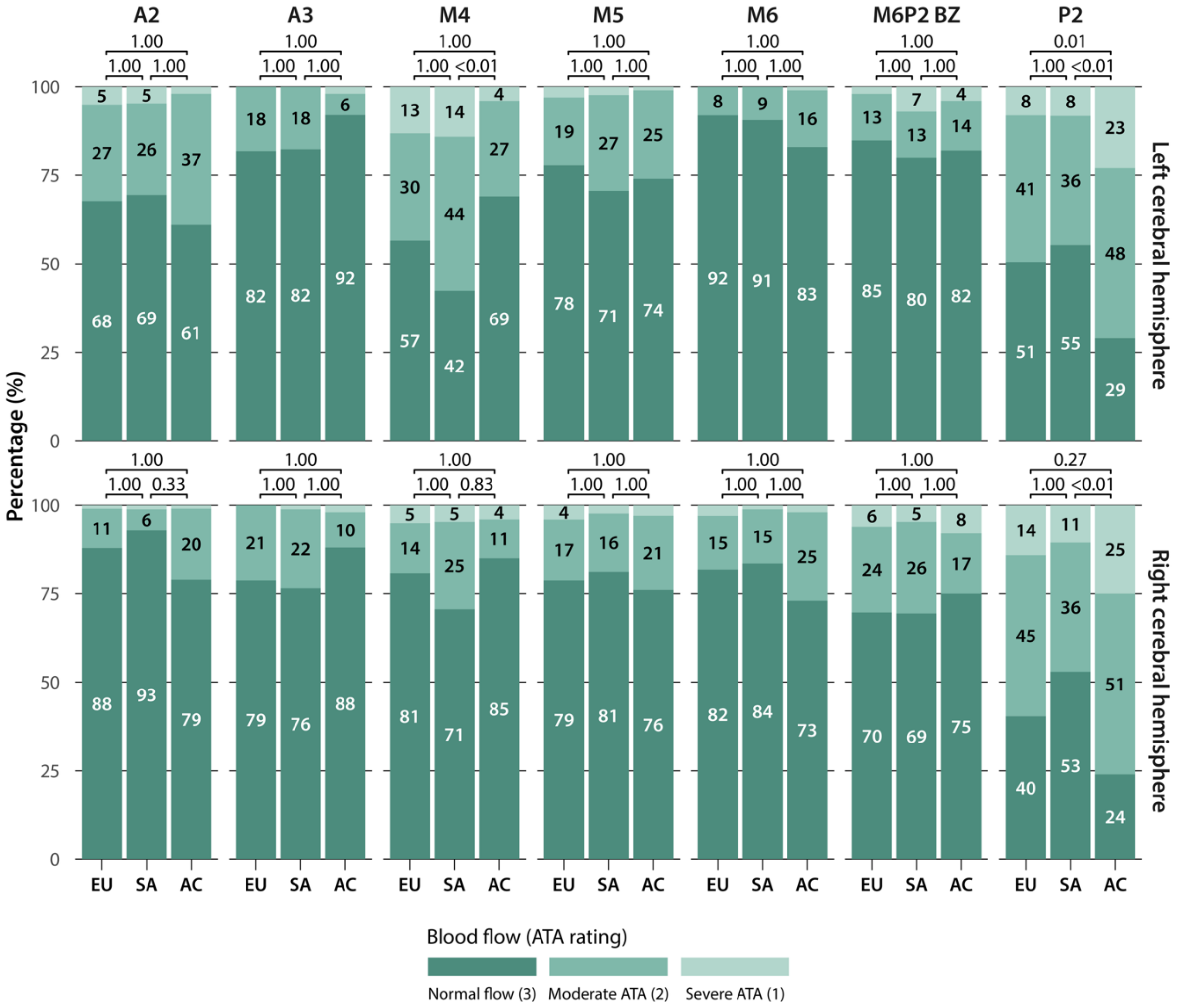

Supplemental Figure 2: Bar plots showing the percentage distribution of ATA ratings for each brain region in the left (upper panel) and right (lower panel) cerebral hemispheres at the level superior to the basal ganglia by ethnicity (White European [EU], N = 99; South Asian [SA], N = 85; African Caribbean [AC], N = 100). Percentages of ATA scores ≥3 are shown (rounded); groupwise comparisons use Holm-adjusted p-values.
